# Microfluidic Cell Cycle Analysis of Spread Cells by DAPI Staining †

**DOI:** 10.3390/mi8020036

**Published:** 2017-01-24

**Authors:** Jing Sun, Jiayu Zhang, Haibo Yang, Gongzhuo Wang, Yanzhao Li, Xuxin Zhang, Qidan Chen, Ming-Fei Lang

**Affiliations:** 1 College of Environmental and Chemical Engineering, Institute of Microanalysis, Dalian University, Dalian 116622, China; yanghaibo@dlu.edu.cn (H.Y.); 18340808897@163.com (G.W.); 2 School of Life Science and Biotechnology, Dalian University, Dalian 116622, China; 18340809524@163.com; 3 Medical College, Institute of Microanalysis, Dalian University, Dalian 116622, China; 4 Affiliated Zhongshan Hospital of Dalian University, Dalian 116622, China; changzhiliyanzhao@126.com (Y.L.); 13842623672@163.com (X.Z.); 5 Department of Chemistry and Pharmacy, Zhuhai College of Jilin University, Zhuhai 519041, China

**Keywords:** microfluidics, cell cycle, spread cell, DNA content

## Abstract

Single-cell cell cycle analysis is an emerging technique that requires detailed exploration of the image analysis process. In this study, we established a microfluidic single-cell cell cycle analysis method that can analyze cells in small numbers and in situ on a microfluidic chip. In addition, factors that influenced the analysis were carefully investigated. U87 or HeLa cells were seeded and attached to microfluidic channels before measurement. Cell nucleic DNA was imaged by 4′-6-diamidino-2-phenylindole (DAPI) staining under a fluorescent microscope and subsequently fluorescent intensities of the cell nuclei DNA were converted to depict histograms for cell cycle phases. DAPI concentration, microscopic magnification, exposure time and cell number were examined for optimal cell cycle analysis conditions. The results showed that as few as a few hundred cells could be measured by DAPI staining in the range of 0.4–0.6 μg/mL to depict histograms with typical cell cycle phase distribution. Microscopic magnification during image acquisition, however, could distort the phase distribution. Exposure time did not significantly affect the cell cycle analysis. Furthermore, cell cycle inhibitor rapamycin treatment changed the cell cycle phase distribution as expected. In conclusion, a method for microfluidic single-cell cell cycle analysis of spread cells in situ was developed. Factors such as dye concentration and microscopic magnification had more influence on cell cycle phase distribution. Further studies will focus on detail differentiation of cell cycle phases and the application of such a method for biological meanings.

## 1. Introduction

Aberrant cell cycle progression is a hallmark of cancer, thus it has been widely studied in cancer research [1]. Recent advances have reinforced targeting cell cycle for cancer treatment. For example, new specific CDK4/6 inhibitors for the treatment of hormone receptor (HR)-positive breast cancers are in phase III trials and palbociclib received fast-track approval by the Food and Drug Administration (FDA) earlier in 2015 [2]. Cyclin dependent kinases (CDKs) 4 and 6 are key regulators for G1-S transition. Previous therapeutic applications of CDK4/6 inhibitors failed due to their toxicity. The new inhibitors are more specific and have much lower IC50 as compared to other CDKs [3]. In glioma, two newly discovered targets (PHF5A and BUB1B/BubR1) involved in cancer-specific cell cycle regulation may prelude novel drugs [4,5]. Knockdown of PHF5A and BUB1B/BubR1 only affected glioma stem cells, but not normal neural stem cells, astrocytes or fibroblasts.

Techniques for cell cycle analysis have been dominated by flow cytometry. Flow cytometry requires a large number of cells (>10^6^) and the cells are in suspension when measured by a flow cytometer, which may disturb the physiology of attached cells [6]. To overcome the limitations of flow cytometry, microfluidics has been introduced to the field of cell cycle analysis. Microfluidic channels hold a tiny amount of media and handles a small number of cells [7]. Microfluidics is also capable of multivariate investigation simultaneously and in high-throughput [8]. This has brought new opportunities for single-cell cell cycle analysis, which is important for the progression of precision or personalized medicine [9,10]. In situ detection of cell cycles by microfluidics has also been explored. Yoo et al. reported cell cycle analysis of cultured HeLa cells in a microfluidic device, demonstrating the feasibility of quantifying DNA contents in adherent cells [11]. DNA contents change during cell division from 2N to 4N and then back to 2N. These changes correspond to different cell cycle phases. In addition, Roukos et al. studied single-cell cell cycle analysis of three adherent cell lines (NIH323, U2OS, and human immortalized skin fibroblasts) in 384-well plates, with the cell DNA stained by 4′-6-diamidino-2-phenylindole (DAPI) or propidium iodide (PI) [12]. The authors validated their protocols by differences in cell numbers and z-stacks (focus height).

Though microfluidics (or 384-well plates) has been applied in single-cell cell cycle analysis of attached cells, no report has systemically presented how the parameters during image acquisition could influence the analysis, such as DNA staining dye concentration, image acquisition magnification, image acquisition exposure time, and cell numbers. In this study, glioma cell line U87 or cervical cancer cell line HeLa was studied by DAPI staining of the cell nucleic DNA for cell cycle analysis. The cells were analyzed in situ in microfluidic channels. Parameters that affect the cell cycle analysis were found to be DAPI concentration and image acquisition magnification. Cell numbers and image acquisition exposure time in this study were not obvious influencing factors.

## 2. Materials and Methods

### 2.1. Design and Fabrication of the Microfluidic Chips

The microfluidic chips were fabricated in the same way as described in our previous study [13]. Briefly, the microfluidic device was fabricated from polydimethylsiloxane (PDMS) (Momentive), which consists of 24 cell culture channels with a channel dimension of 8 mm (*l*) × 1 mm (*w*) × 120 μm (*h*). The PDMS was then attached onto a poly-l-lysine (PLL)—coated glass slide. Cells and culture media were introduced into the microfluidic channels through an inlet and wastes were removed from an outlet.

### 2.2. Cell Culture in the Microfluidic Channels

Glioma cell line U87 and cervical cancer cell line HeLa were purchased from the Cell Bank of the Chinese Academy of Sciences, Shanghai, China. The cells were cultured in Minimum Essential Medium (MEM) (Thermo Fisher Scientific Inc., Rockford, IL, USA), supplemented with 10% fetal bovine serum (Zhejiang Tianhang Biotechnology, Hangzhou, China), 1 × non-essential amino acids (Thermo Fisher Scientific), 1 × sodium pyruvate (Thermo Fisher Scientific), and 1 × penicillin/streptomycin (Thermo Fisher Scientific) in a 5% CO_2_, 37 °C incubator. After the cells (250–500 cells/μL) were suspended in the culture media and loaded into the microfluidic channels, the microfluidic chip was briefly centrifuged for the cells to attach. The cells were cultured overnight before further analysis. For cell inhibitor treatment, a final concentration of 20 nM rapamycin (Meilune Biotech, Dalian, China) was added into the culture medium and the cells were incubated for the indicated time.

### 2.3. DAPI Staining

Cells were first fixed by 4% paraformaldehyde (Aladdin, Shanghai, China) in the microfluidic channels at room temperature for 10 min, washed with phosphate buffered saline (PBS) three times, permeabilized by 0.3% Triton X-100 (Aladdin) for 10 min, washed with PBS again three times, and finally stained by 4′,6-diamidino-2-phenylindole (DAPI) (Meilune Biotech) for 15 min.

### 2.4. Image Acquisition and DNA Content Analysis

Images of the DAPI stained cells were obtained with a Nikon Eclipse Ti inverted fluorescent microscope with a charge-coupled device (CCD) camera. Each image was focused manually. DAPI fluorescent intensities in individual cells were quantified by MetaMorph (Molecular Devices).

### 2.5. Histogram Generation

Integrate intensities of the DAPI staining were converted to frequency count with an interval of 60 by Origin 8.0 (OriginLab, Northampton, MA, USA). The bin center (*x* axis) and count (*y* axis) were plotted to generate histograms to reflect the cell counts at relative integrate intensities. Cell counts (*y* axis) were normalized by the total cell numbers to obtain the percentage of cells at each cell cycle phase. Integrate intensities (*x* axis) were normalized to obtain a relative DNA content. Manually set gates in the histograms were used to calculate the percentages of cells within G1, S and G2/M phases using the R software as described in Roukos et al. [12].

### 2.6. Statistical Analysis

Data were presented as mean ± standard deviation (SD) from three independent analyses. One-way analysis of variance (ANOVA) was used to calculate statistical significance by GraphPad Prism 6. *p* < 0.05 was considered as statistically significant.

## 3. Results and Discussion

**Figure 1:**
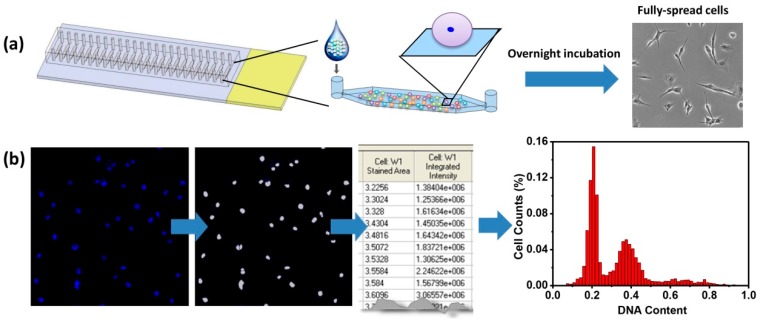
Schematic of microfluidic cell cycle analysis. (**a**) Image of a microfluidic chip with dye-filled channels, one of which is enlarged to illustrate cells seeding. After overnight culture, cells are fully spread and ready for cell cycle analysis; (**b**) Fluorescent images of nucleic DNA stained by 4′-6-diamidino-2-phenylindole (DAPI) are obtained and fluorescent intensities of the nuclei are converted to draw a histogram against cell numbers.

The microfluidic device was fabricated as shown in Figure 1a. After introducing single cell suspensions into the channels, the cells were centrifuged briefly and cultured overnight. These procedures were to ensure cell attachment as well as cell spreading. By using fully-spread cells, adherent cells were minimally disturbed before cell cycle analysis. However, in flow cytometry, adherent cells are detached before analysis, which may remove or decrease some surface antigens that can cause misinterpretation of the actual cell conditions. Next, the cell nucleic DNA was stained by DAPI and fluorescent images were obtained. Fluorescent intensities of each nucleus were converted to depict histograms against cell numbers (Figure 1b). Similar to flow cytometry analysis of cell cycles, the histograms could identify cell cycle phases. In phase G0/G1, DNA is not replicated and the DNA content is 2N. During S phase, DNA is being replicated and the DNA content is dynamic in the cell, in the range of 2N–4N. G2 is the final gap in cell cycle before cell division happens. Till the two daughter cells are fully separated, the DNA content in a cell is 4N. Thus, G0/G1 phases have 2N of DNA, S phase has 2N–4N of DNA, and G2/M phases have 4N of DNA. In this regard, seeding density is an important factor for in situ single-cell cell cycle analysis, since overcrowded cells may result in poor resolution of cell clusters by the image quantification software, which relies on the recognition of individual cells. For example, when two cells are too close, they may be considered as one cell and this leads to incorrect nucleic DNA content quantification. In our previous study, seeding density was optimized and the same density was used in the current study [13].

**Figure 2:**
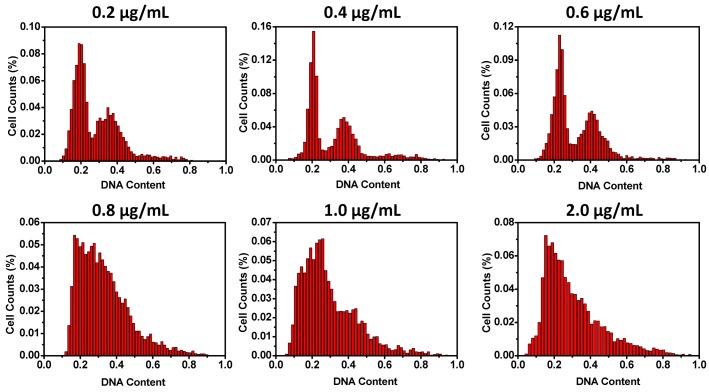
Influence of DAPI concentrations on cell cycle analysis. Histograms of fluorescent intensities of nucleic DNA against cell numbers were depicted at different DAPI concentrations (0.2–2.0 μg/mL). An optimal DAPI concentration range (0.4–0.6 μg/mL) was determined, under which two peaks representing 2N and 4N nucleic DNA could be easily distinguished.

In order to search for optimized conditions for microfluidic cell cycle analysis of spread cells, a glioma cell line U87 was utilized. U87 cells are adherent and have a typical flat and protrusive morphology. After overnight culture, the cells were attached to the bottom of the channels and made fully spread. By DAPI staining, DNA contents in the cell nuclei could be visualized under a fluorescent microscope. Since this study was to assess the possibility of DAPI staining of spread cells on the measurement of cell cycles, DAPI concentrations were first optimized. With the six DAPI concentrations examined, DAPI concentrations higher than 0.6 μg/mL produced histograms lacking the characteristic two-peak distribution of cell cycle phases. DAPI concentrations no more than 0.6 μg/mL resulted in flow cytometry like histograms (Figure 2). An amount of 0.4 μg/mL of DAPI was used for the following experiments.

**Figure 3:**
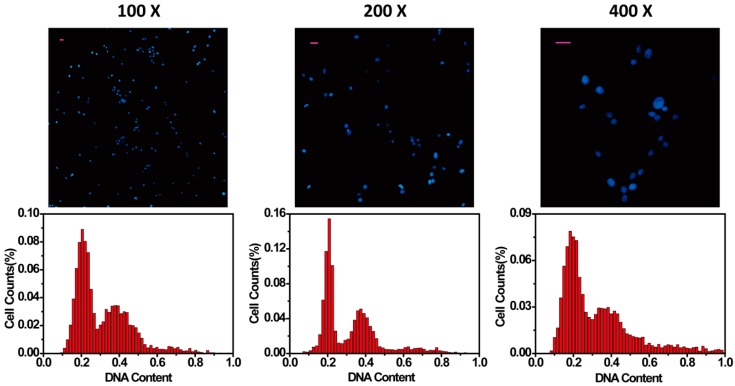
Influence of microscopic magnification on cell cycle analysis. Histograms under microscopic magnification of 100×, 200×, and 400× were depicted; showing that 200× magnification was the most suitable for cell cycle analysis. Scale bars represent 25 μm.

Secondly, the magnification effect of cell nuclei images was explored. Regular microscopic magnifications of 100×, 200×, and 400× resulted in obvious differences in the distribution of the cell cycle phases (Figure 3). Magnification of 200× showed the best distribution as compared to previous results in this study.

**Figure 4:**
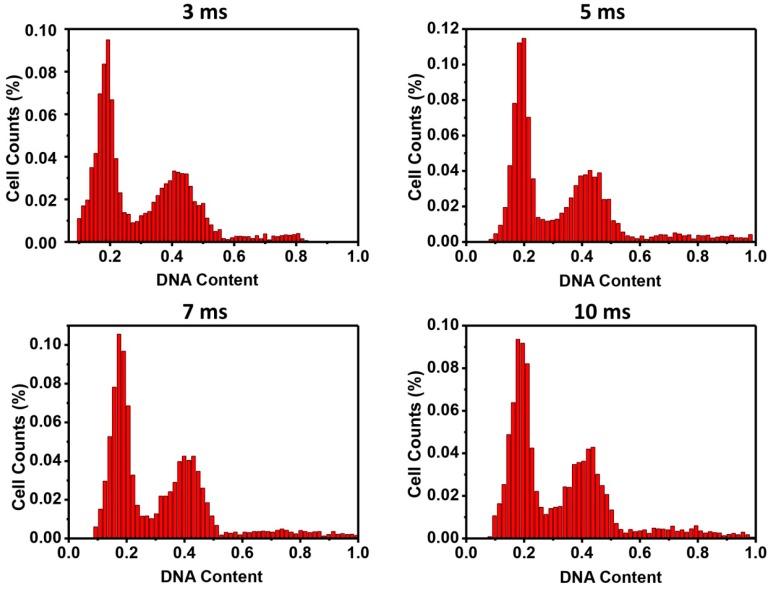
Influence of exposure time on cell cycle analysis. Fluorescent nucleic DNA was exposed between 3 and 10 ms and histograms of the fluorescent intensities were depicted against cell numbers, which showed no major difference.

Thirdly, exposure time could also affect the reading of fluorescent intensities during fluorescent image acquisition. By exposing the DAPI stained cells with different times from 3 to 10 ms, histograms with a typical cell cycle phase distribution were generated, showing no obvious difference (Figure 4). The exposure time then was determined first by the auto-expose function of the image analysis software, mostly about 5 ms in our experimental settings. This allowed precise and fast scanning of the fluorescent images. In order to calibrate the exposure, intensity surface plots showing individual fluorescent intensities of each DAPI stained cell nucleus were examined (Supplementary Figure S1). The intensity peaks from 3 to 10 ms of exposure demonstrated that DAPI stained cell nucleic DNA content was within the detection limits of the system. No over-exposure (flat peaks) or under-exposure (missing peaks) was observed. The three-fold exposure time change in this study also represented a relatively wide window for adjusting exposure in the image acquisition without affecting the quality of the analysis. The short 3–10 ms exposure time was applied in our experimental settings, because a new halogen lamp was used. When a halogen lamp is used for a while, the exposure time can increase significantly (e.g., to a few hundred milliseconds). In such a case, the image acquisition time would be extremely extended, since many pictures are taken for image analysis. Longer exposure will also increase background noise that can result in false-positive signals. Background subtraction must be considered when longer exposure time is used. From our results, exposure time around the 'auto-expose' should not affect data analysis.

**Figure 5:**
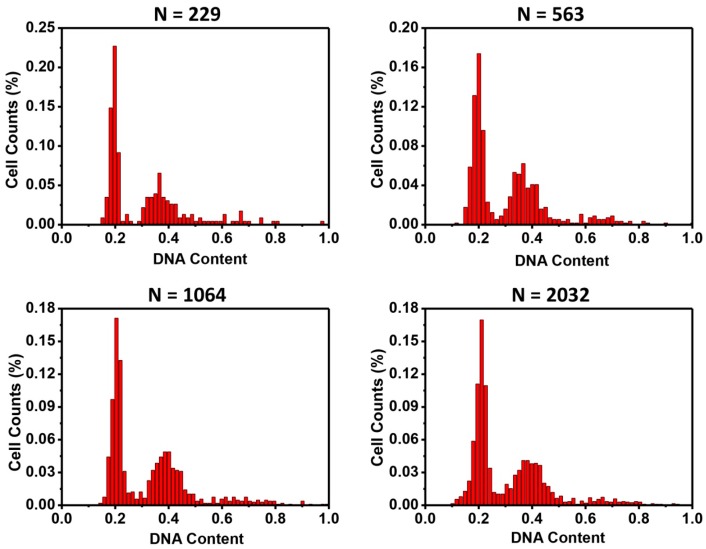
Influence of total cell numbers on cell cycle analysis. With the numbers of cells tested (229–2032), all resulted in similar histograms of cell cycles.

Fourthly, the numbers of cells required in the cell cycle analysis were quantified. After images of DAPI stained nuclei were acquired, one image was randomly divided into several sub-regions, with different numbers of cells in each sub-region. Then, cell cycle histograms were generated (Figure 5). With the numbers of cells analyzed, all could lead to typical two-peak cell cycle phase distribution. However, narrower and more centered peaks were seen when cell numbers increased. These results demonstrated that the microfluidic cell cycle analysis method could use much fewer cells (as few as a few hundred). For some types of studies, in which cell numbers are limited, microfluidic cell cycle analysis is a valuable tool.

**Figure 6:**
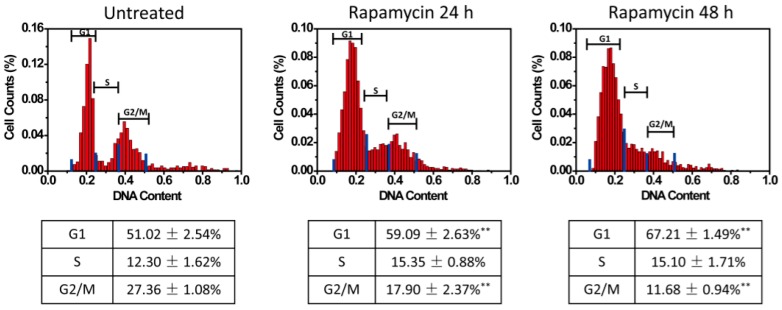
Rapamycin induced G1 arrest of U87 cells. U87 cells were treated by rapamycin (20 nM) and the cell cycles were measured. Different cell cycle phase distribution was observed: G1 51.02% ± 2.54%, S 12.30% ± 1.62%, G2/M 27.36% ± 1.08% for the untreated cells; G1 59.09% ± 2.63%, S 15.35% ± 0.88%, G2/M 17.90% ± 2.37% at 24 h after the treatment; G1 67.21% ± 1.49%, S 15.10% ± 1.71%, G2/M 11.68% ± 0.94% at 48 h after the treatment. The blue bars in the histograms were manually set gates for calculating cells in the cell cycle phases. Data from three independent analyses were presented as mean ± SD. ** *p* < 0.01 (as compared to the same cell cycle phase in the untreated group).

Lastly, the U87 cells were treated with rapamycin. Rapamycin is an mTOR inhibitor and can induce G1 arrest in glioma cells [14,15]. When treated with rapamycin, more cells accumulated at the G1 phase (Figure 6). The untreated cells showed a cell cycle phase distribution of G1 51.02% ± 2.54%, S 12.30% ± 1.62%, and G2/M 27.36% ± 1.08%. More cells shifted to the G1 phase at 24 h (G1 59.09% ± 2.63%) and 48 h (G1 67.21% ± 1.49%) after rapamycin treatment (*p* < 0.01, as compared to the G1 phase in the untreated group). The percent of cells in the G2/M phases dropped to 17.90% ± 2.37% at 24 h and G2/M 11.68% ± 0.94% at 48 h ((*p* < 0.01, as compared to the G2/M phase in the untreated group), and in the S phases showed less alteration 15.35% ± 0.88% at 24 h and 15.10% ± 1.71% at 48 h (*p* > 0.05, as compared to the S phase in the untreated group). These results also validated the single-cell cell cycle analysis in dynamic culture conditions that influenced cell cycle phase distribution. To further validate the analysis, HeLa cells were stained by Hoechst 33258 (a DNA dye) and cell cycle phases could also be resolved (Supplementary Figure S2).

Microfluidic cell cycle analysis of spread cells is an emerging field that represents new opportunities for studying more accurate and systemic characteristics in a cell. When some cell cycle phase differentiation markers are investigated together with the DNA content analysis, cell activities in cell cycle phases are linked to more narrower windows during cell division. For example, Ctd1 is a G1 specific marker that achieves highest expression levels before the cell enters S phase, when Ctd1 expression decreases dramatically [16,17]. Our previous results have demonstrated the possibility of detecting multiple proteins at single cell levels [13]. Therefore, it is plausible that DNA content analysis and simultaneous multiple protein staining will enable an expanding role of microfluidics in cell cycle analysis.

## 4. Conclusions

Our study systemically examined the influence of different factors (DAPI concentration, magnification, exposure time, and cell number) on the microfluidic cell cycle analysis. The major factors that can affect the results are DAPI concentration and magnification. Exposure time and cell number have less influence. However, the analysis is a combined methodology that requires adaptation and validation in each individual study.

## Supplementary Materials

The following are available online at www.mdpi.com/2072-666X/8/2/36/s1, Figure S1: Fluorescent intensities at different exposure times, Figure S2: Single-cell cell cycle analysis of Hela cells by Hoechst staining.
