# Supplementary material for: Microfluidic Cell Cycle Analysis of Spread Cells by DAPI Staining
Source: Micromachines (Basel). 2017 Jan 24;8(2):36. doi: 10.3390/mi8020036 (PMC6189792; doi:10.3390/mi8020036)
Supplement: Supplementary file 1 [file micromachines-08-00036-s001.pdf]

# Supplementary Materials: Microfluidic Cell Cycle Analysis of Spread Cells by DAPI Staining

**Jing Sun, Jiayu Zhang, Haibo Yang, Gongzhuo Wang, Yanzhao Li, Xuxin Zhang, Qidan Chen and Ming-Fei Lang**

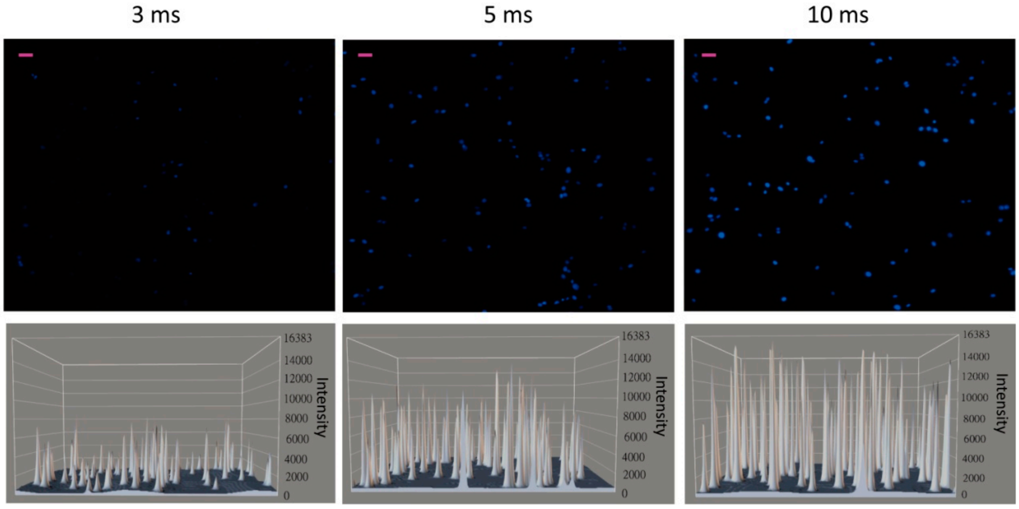


**Figure S1.** Fluorescent intensities at different exposure times. Upper panel: representative images showing 4′-6-diamidino-2-phenylindole (DAPI) stained cell nuclei of U87 glioma cells under 200× magnification (scale bars = 25 μm); Lower panel: corresponding intensity surface plot showing individual fluorescent intensities of each DAPI stained cell nucleus. Note that the intensities at different exposure times were within the detection limits of the microscope and the intensities had sharp, instead of flat (indicating saturation), peaks. The highest intensity that the image acquisition system could detect was 16,383, which was shown on the Intensity axis of the surface plots.

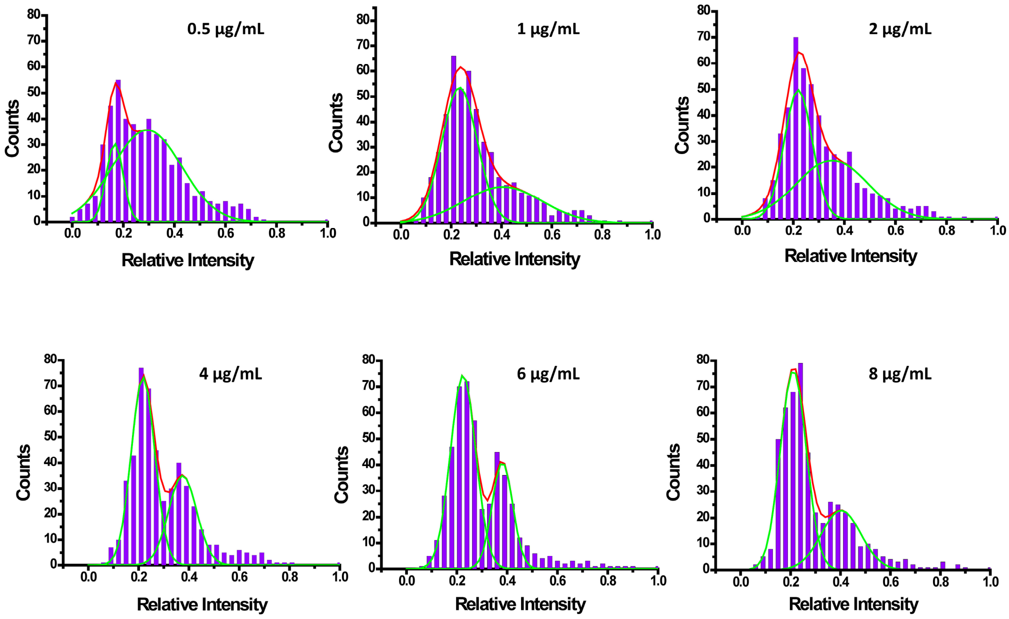


**Figure S2.** Single-cell cell cycle analysis of Hela cells by Hoechst staining. Hoechst 33,258 was added into the culture medium of Hela cells. Different concentrations of the Hoechst dye were tested (0.5–8 μg/mL). Typical cell cycle phase distribution was observed at 4, 6, and 8 μg/mL of the Hoechst dye. In each measurement, 1000 cells were used, the exposure time was 5 ms, and a magnification of 200× was applied.
